# Cholera Infantum

**Published:** 1884-07

**Authors:** Ad. Lippe

**Affiliations:** Philadelphia


### CHOLERA INFANTUM.

AD. LIPPE, M. D., PHILADELPHIA.

*Cholera Infantum*, or, as this form of disease is generally termed, "summer complaint," comprises all the various diseases of the digestive organs and brain with which children are attacked during the summer, and most frequently during dentition during their second summer. The various forms of diseases of the digestive organs are those attacking the stomach as its principal seat, as catarrh, acidity, inflammation, ulceration, or softening of it, or the intestines alone are the seat of the disease, as an erithematous inflammation, catarrh, excoriations, and ulceration.

The disease often appears in different forms, at different seasons, and in different localities.

The brain is very frequently the seat of the disease from the very inception of it, and the erroneous idea that a later stage of the disease itself develops the various cerebral symptoms is only a proof that the first observations of the state of the patient's disturbed health were made inaccurately, and that the cerebral symptoms had been entirely overlooked. The most frequent brain disturbance, from the very beginning of the disease, is hydrocephaloid.

If the observing healer has found the cerebral symptoms (dilated pupils, hot head, cold extremities, drowsiness) present in a child during the hot weather and the prevalence of cholera infantum, he may avert all further anxieties (especially if the child also vomits) by administering a single dose of Belladonna.

The knowledge of the seat of the disease, its nature, its name, or a knowledge of the stage in which we find the disease, does not indicate a particular treatment, or indicate the truly curative remedy; but this knowledge is nevertheless necessary, for it facilitates the examination of the sick, and it enables the physician to classify the symptoms obtained, and to consider as most important in each individual case the symptoms indicating the progress of disease in this or the other locality, and the changes or suppression of one or the other functions of organs. As an illustration of these propositions, let us turn to a child supposed to suffer from epidemic cholera infantum; we are informed that the child has diarrhœa since midnight, but does not give signs of pain; it lies quiet, its eyes are only half closed, the anterior fontanel is elevated, the face is pale, the wrists and feet are cold, and upon further inquiry we learn that the child has not passed any urine since the previous evening; the abdomen is flabby, not hot. It would be useless, in such a case, to select the remedy guided mostly by the nature of the evacuations. Here we are presented with a decided case of hydrocephaloid, a case of great gravity, possibly to end fatally within one, or, at least, a few days; and we further know that should the patient pass urine within a few hours after the administration of the truly homœopathic remedy (Sulphur in this case), the recovery becomes a certainty, and probably without any further medication. Another child has cholera infantum, and cries most persistently, has done so all night, is cutting teeth, and the distressed mother says this screaming has lasted all night; we have to carry the child all the time to pacify it; it has frequent green discharges from the bowels, preceded by an increase of pain, causing it to draw up its knees to the abdomen; the abdomen is hot, the thirst incessant; we are sure there is nothing the matter with the child's brain, but the seat of the inflammatory disease is in the small intestines. A dose of Chamomilla will soon quiet the child.

In the first case the brain symptoms, with the concomitant suppression of the urinary discharge, stand foremost; in the second case the intestinal symptoms, with the concomitant restlessness and the desire to be carried, stand most prominent.

Knowing that the gravest cases of cholera infantum appear without any previous indisposition, without any precursory diarrhœa, probably with no other warning than a little more sleepiness of the otherwise, to all appearances, well child, and that in just such cases all depends on the proper choice of the first remedy, we must be prepared beforehand to choose right, and administer the remedy according to the Homœopathic Law of Cure.

It has been proposed to begin the treatment of these grave cases of cholera infantum, having their origin in a disturbed condition of the brain, by administering Aconite and Bryonia in alternation. As this proposition is a violation of all and every fundamental principle of our school, the result will be a failure to cure. First and foremost, the character of the disease, its locality, or its kind, can never serve as a guide to our therapeutic action; much less can the administration of two entirely differently acting drugs, as are Aconite and Bryonia, be followed by salutary results; either one or the other can stand in the proper relation as a therapeutic agent under the Law of the Similar, never both, and why, then, not adhere to the Law, and administer the Simillimum?

The therapeutics include also the dietetics, and in cholera infantum it becomes very important to see to it that the proper nourishment is given to the children. The better the dietetics of a child have been understood, and the more proper the nourishment has been from its birth, the less liable will it be to be attacked violently by the ordinary diseases of children during the hot weather. There are general dietetic rules for children laid down in the books, and it is all well to know them, but they lead to generalizations; each individual child wants its own individual diet, adapted to its own individual constitutional condition. The administration of crude substances supposed to be wanting in the organism is based on "materialism;" the substances so wanting, or supposed to be wanting, can at best only be supplied by food containing them in a greater proportion than its ordinary nourishment did. The instinct of children will very frequently indicate the requisite nourishment, which then should never be withheld, if it is even contrary to speculative science. In properly nourished children we will rarely ever find a bad case of cholera infantum, and the more we have studied carefully the proper diet of each individual child under our care, the less will they be liable to diseases of the digestive organs. Many cases of children come under the treatment of the physician which he has never seen before, and the more general experience he has gained about the proper diet of children, the easier will he be able to detect what mistakes have been made in each individual case, and he will at once endeavor to correct the erroneous diet.

The erroneous but generally accepted notion that children should be nursed during the second summer on account of the prevalence of cholera infantum during that season causes more cases and is the frequent cause of the great mortality in that disease. There are nine months of gestation, and exactly nine months of lactation (nursing and feeding by the mother's milk). The appearance of the teeth is the first indication that farinaceous food is wanted, and it must not be withheld, and as different children cut their teeth earlier or later during the first nine months, the farinaceous food should be given as it is needed.

All the dietetics being properly attended to, it remains to find in each individual case the corresponding similar remedy. In grave cases, the choice of a remedy must be made at once, as delay is attended by great danger. It is the aim of these short pages to give characteristic symptoms, and a concise description of frequently occurring combinations of symptoms in this form of disease, with their correspondingly similar remedy.

We shall first give the most frequently indicated remedies, and then those less often called for.

Apis mel.—The child is inclined to stupor, out of which it starts with a loud, shrill scream. The eyes have a reddish tint. The head is hot. The tongue is dry, but thirst is but seldom present. The skin is dry, the hands at times cold and blue. Suppression of urine. The abdomen is tender to pressure. The diarrhœa is worse in the morning, always mixed with mucus, sometimes very offensive or involuntary, or containing flakes of pus.

Belladonna.—The child lies in a stupor; it frequently starts up suddenly in his sleep; when awake it is angry and violent. The head is hot, and is often rolled from side to side. The face is generally purple, red, and hot, or very pale and cold. The tongue is red on the edges, or coated whitish yellow, or has two white strips of coating extending down on both sides of the tongue. Thirst moderate. Pulse very frequent, small, and hard, occasionally full. Hands and feet cold; the hotter the head is, the colder are the feet. The abdomen is hot. The stools are clay color or green, or consist of white or granular yellow slimy mucus, and very frequent.

CHAMOMILLA.—The child is exceedingly peevish; the gums are very hot, the cheeks are red, at times only one cheek; the child wants to be carried all the time; has attacks of colic, draws its knees up, and seems to be relieved for a short time after a passage from the bowels. Vomiting of food and sour mucus. The stools are green, or green mucus at times mixed with white mucus or chopped; the discharges are hot, excoriating the parts, frequent, sometimes smelling like rotten eggs.

CROTON TIGLIUM.—The child has a stool as often as it is fed or nurses. The discharge is sudden, noisy, and violent, consisting generally of yellow water.

IPECACUANHA.—Diarrhœa and vomiting. Vomiting of food and drink as often as one drinks, or vomiting of green mucus. Much nausea, with pale face and oppressed breathing. Stools consist of green mucus, or are bloody or fermented.

NATRUM SULPHURICUM.—Frequent attacks of violent colic, with rumbling in the abdomen, relieved by the violent discharge of yellow water with large quantities of flatus. The stools are more frequent during the morning hours, after the child has been taken up and is moved about, like Bryonia.

PODOPHYLLUM PELT.—Drowsiness or restless sleep, with grinding of the teeth or rolling of the head. Vomiting of frothy mucus, green, or of food. The diarrhœa is worse in the morning, and the discharges are more frequent at night than during the day. Stools green, watery, or mixed with mucus, or like chalk; profuse and painless. During and after stool, prolapsus ani. During dentition also catarrhal cough and catarrh of the chest. Cramps of the feet, calves, and thighs.

SULPHUR.—The disease generally begins after midnight; diarrhœa and vomiting; the discharges from the bowels are generally watery, green, and involuntary; they sometimes smell sour, at other times they are very offensive; vomiting is frequent, often smelling sour (like Calc. c.), with cold perspiration on the face (Veratr., cold perspiration on the forehead). The face is pale, the fontanels open, hands and feet cold the very first morning; the child lies in a stupor with its eyes half open; not much thirst and entire suppression of urine. The child does not scream out violently as under Apis, or roll his head as under Belladonna. In such a case as above described one single dose of Sulphur will suffice to re-establish the urinary secretions and cause the child to sit up again and take food.

Aconitum nap. is seldom indicated, and then only at the beginning of the disease, especially when it has been caused by a check of perspiration, mostly during the night, when the weather has changed from extreme heat to cold. The child is excessively agitated and restless, pulse very frequent and hard, abdomen very hot; much thirst; the discharges are watery and contain bloody mucus.

Arsenicum.—Diarrhœa and vomiting; much thirst for cold water, but everything the child drinks is thrown up at once; hot skin, great restlessness; the child continuously tosses about, changes its position, and cries incessantly. Stools watery and very offensive, or black fluid, or dark, thick green mucus; very great weakness and emaciation.

Benzoic. acid.—If, during an attack, the urinary discharges become very scanty, and if the urine has a very pungent, strong smell, and if the urine easily becomes turbid.

Bismuth.—Diarrhœa and vomiting. The vomiting prevails; all food and drink is thrown up at once; the abdomen is bloated, the face is pale, blue rings under the eyes. (Compare Creosote.)

Bryonia.—The attacks return as soon as the weather becomes very hot, and are relieved on cool days. (Aconite and Dulcamara have the reverse.) Vomiting of bile, tongue coated yellow, thirst, not frequent, but drinking of large quantities (Aconite has the reverse); abdomen hot, the child does not want to be moved (Aconite has the reverse); every motion causes pain in the abdomen and a discharge from the bowels. Worse in the morning when beginning to be moved.

Calcarea carbonica.—Open fontanels; stools gray—like clay, smelling *sour;* vomiting of food, and especially milk, *sour;* profuse perspiration on the head during sleep; swollen, distended abdomen (Sacchar. off.); urine clear (Benz. ac. has turbid urine), is passed with difficulty, and has a strong pungent, fetid odor.

Carbo veget.—Diarrhœa; stools very putrid or bloody; face pale or greenish; the gums recede from the teeth and bleed easily; abdomen distended; emissions of large quantities of flatus; skin cold; tongue and breath cold; voice hoarse or lost.

China.—Painless watery diarrhœa, yellow or blackish or of indigested food; worse after eating (Ferrum has diarrhœa while eating), and worse at night and after eating fruit, with much tendency to perspire.

COLOCYNTHIS.—Diarrhœa with violent colic before, during, or after the stool, compelling the child to bend double, which seems to give relief (the colic of Belladonna is relieved by hard pressure across the abdomen; that of Rhus tox. is relieved by lying on the abdomen).

CREOSOTE.—Diarrhœa with vomiting; the continuous vomiting and straining to vomit predominates; the child resists the tightening of anything around the abdomen, which increases its restlessness and pain; much thirst; gums hot; coldness of the hands and feet. (Compare Bismuth.)

IRIS VERS.—Diarrhœa and vomiting; vomiting of food, bile, or of a very sour fluid; profuse, frequent, watery stools. Tympanitis.

NATRUM MUR.—Watery diarrhœa with colic; incessant thirst with nausea; emaciation beginning at or principally on the neck; abdomen bloated.

NITRIC. ACIDUM.—Diarrhœa, green, mucous or bloody, or putrid; putrid smell from the mouth; copious flow of saliva; ulcers in the mouth and on the tongue.

PAULLINA SORBILIS.—Green profuse stools, *inodorous.*

PETROLEUM.—Diarrhœa *only* during the day.

PHOSPHORUS.—Diarrhœa and vomiting; desire for cold water, which is thrown up as soon as it becomes warm in the stomach; diarrhœa is worse in the morning; stools consist of green mucus, brown fluid, white mucus, or containing little grains like tallow.

SILICEA.—Fontanels open; much perspiration on the head; great thirst; emaciation; rolling of the head; suppressed urinary secretions; watery, very offensive stools. (Calc. c. has sour-smelling stools.)

SULPHURIC ACID.—Frequent, large, watery, very offensive evacuations, with aphthæ and great irritability.

VERATRUM ALBUM.—Diarrhœa and vomiting; great weakness; vomiting of frothy substance; profuse watery diarrhœa, with flakes; during stool cold perspiration on the forehead; pale face; cold hands; voice weak or hoarse; suppression of urine,

If marasmus follows a protracted case of cholera infantum we have two great principal remedies to stay its progress and cure the patient.

SARSAPARILLA.—Great emaciation; the skin lies in folds; the face is shriveled; aphthæ on the tongue and on the roof of the mouth.

IODINE.—The child has an inordinate appetite, but nevertheless continues to emaciate.

If effusions on the brain have taken place, then we may resort to Digitalis, Helleborus, Hyosciamus, Opium, Zinc, according to their respective indications.

These general indications will enable the practitioner to find the proper remedy in many cases, especially in cases requiring prompt and unhesitating prescriptions. The variety of cases is so great that it is utterly impossible to give a proper prescription for all and every variety of cases of cholera infantum or any other disease.

